# Signs of early cellular dysfunction in multiple system atrophy

**DOI:** 10.1111/nan.12661

**Published:** 2020-09-17

**Authors:** M. Herrera‐Vaquero, A. Heras‐Garvin, F. Krismer, R. Deleanu, S. Boesch, G. K. Wenning, N. Stefanova

**Affiliations:** ^1^ Division of Neurobiology Department of Neurology Medizinische Universitat Innsbruck Innsbruck Austria; ^2^ Institute of Neuroscience Medical University of Innsbruck Innsbruck Austria

**Keywords:** multiple system atrophy, induced pluripotent stem cells, mitochondria, high resolution respirometry, α‐synuclein, oxidative stress

## Abstract

**Aims:**

Multiple system atrophy (MSA) is a fatal neurodegenerative disease that belongs to the family of α‐synucleinopathies. At post mortem examination, intracellular inclusions of misfolded α‐synuclein are found in neurons and oligodendrocytes and are considered to play a significant role in the pathogenesis. However, the early steps of the disease process are unknown and difficult to study in tissue derived from end‐stage disease.

**Methods:**

Induced pluripotent stem cells (iPSCs) were generated from patients’ and control skin fibroblasts and differentiated into NCAM‐positive neural progenitor cells (NPCs). The mitochondrial morphology and function were assessed by immunocytochemistry and high resolution respirometry. The ability to cope with exogenous oxidative stress was tested by exposure to different doses of luperox. The expression of α‐synuclein was studied by immunocytochemistry.

**Results:**

We identified increased tubulation of mitochondria with preserved respiration profile in MSA‐derived NPCs. Exposure of these cells to exogenous oxidative stress even at low doses, triggered an excessive generation of reactive oxygen species (ROS) and cleavage of caspase‐3. MSA‐derived NPCs did not present changed levels of *SNCA* gene expression nor intracellular aggregates of α‐synuclein. However, we identified disease‐related translocation of α‐synuclein to the nucleus.

**Conclusions:**

Our results show early cellular dysfunction in MSA‐derived NPCs. We identified changes in the redox homeostasis which are functionally compensated at baseline but cause increased susceptibility to exogenous oxidative stress. In addition, nuclear translocation of α‐synuclein in MSA‐derived NPCs supports an early cellular stress response which may precede the neurodegenerative process in this disorder.

## Graphical abstract

Neural progenitors derived from iPSCs of patients with multiple system atrophy show changes in the mitochondrial morphology, relocation of α‐synuclein to the nucleus, and increased susceptibility to exogenous oxidative stress. The findings suggest early cellular dysfunction preceding α‐synuclein aggregate formation in this disorder.

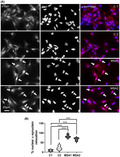

**Abbreviations**
CCCP: carbonyl cyanide m‐chloro phenyl hydrazone
ETS: electron transfer system
FCF: Flux control factors
FCR: Flux control ratios
GCIs: glial cytoplasmic inclusions
GNIs: glial nuclear inclusions
GWAS: Genome‐wide association study
hESCs: human embryonic stem cells
iPSCs: induced pluripotent stem cells
MSA: multiple system atrophy
NCAM: Neural cell adhesion molecule
NCIs: neuronal cytoplasmic inclusions
NNIs: neuronal nuclear inclusions
NPCs: neural progenitor cells
PSA‐NCAM: Polysialylated‐neural cell adhesion molecule
qRT‐PCR: quantitative real‐time polymerase chain reaction
RCR: respiratory acceptor control ratio
ROS: reactive oxygen species
ROX: residual oxygen consumption
SeV: Sendai virus
SUIT: substrate‐uncoupler‐inhibitor titrations

## Introduction

Multiple system atrophy (MSA) is a fatal and rapidly progressive neurodegenerative disorder. It is a rare disease and the average prevalence is 7.8 per 100,000 among persons older than 40 years of age [1]. MSA is commonly diagnosed in the sixth decade of life with variable combination of autonomic dysfunction and motor symptoms, including Parkinsonism and cerebellar ataxia [2, 3]. The pathological hallmark of the disease is α‐synuclein positive cytoplasmic inclusions in oligodendroglia (glial cytoplasmic inclusions (GCIs)), however neuronal cytoplasmic and neuronal/glial nuclear inclusions (NCIs, NNIs, GNIs) are also common, while Lewy bodies are identified in only 10% of patients [4, 5]. In comparison to Parkinson’s disease, the most common α‐synucleinopathy, MSA has a more rapid progression and death generally occurs 9 years after the diagnosis [3, 6, 7, 8].

The aetiology of MSA remains largely elusive. MSA seems mostly sporadic as proposed by a genome‐wide association screening [9]. Population‐specific point mutations in the *COQ2* loci in few cases [10, 11, 12, 13, 14] have suggested a leading pathogenic role of dysfunctional mitochondria in MSA. Other studies have supported the role of mitochondrial deficits in the pathogenesis of MSA regardless of the genotype [15]. A recent epigenome‐wide association study in MSA has further implicated pathways relevant to neurodegeneration, including those related to neuroinflammation and mitophagy, in the pathogenesis of the disease [16].

Disease mechanisms have been further addressed in studies applying post mortem tissue, animal and cellular models [17, 18, 19, 20, 21]. Unfortunately, post mortem analyses provide only a limited time‐window into the end‐stage of the disease. Alternatively, the existing animal and cellular models (cell lines and primary cultures) which are based on the overexpression of α‐synuclein [18, 19, 22, 23] or α‐synuclein propagation [24, 25, 26] are not in the position to identify the initial cellular changes that may contribute to the specific neuronal and oligodendroglial pathology in MSA. The expression of *SNCA*, the gene encoding α‐synuclein, is generally not changed in MSA brains [27]. The putative prion nature of MSA as proposed in experimental studies is challenged by the fact that endogenous wild‐type α‐synuclein is insufficient to propagate MSA‐derived α‐synuclein pathology and mutated α‐synuclein is needed as a template [24]. Strain differences of α‐synuclein in the different synucleinopathies [28, 29] have been recently proposed to contribute to the more aggressive progression of MSA [30], however their origin and role in triggering disease remain unclear. Pathological and experimental observations have further suggested that early changes in oligodendrocytes associated with relocation of the myelin‐related protein p25α may precede α‐synuclein pathological accumulation in these cells [31, 32, 33]. These data pose the hypothesis that cellular dysfunction preceding α‐synuclein pathology may be present in MSA, however examining the earliest stages of the disease remains challenging.

Induced pluripotent stem cell (iPSC) technologies allow dynamic cellular studies in patient‐specific lines, which may provide insights into the early cellular dysfunction featuring the MSA background. Here, we describe for the first time the use of iPSC‐derived neural progenitor cells (NPCs) to study disease‐related cellular dyshomeostasis in MSA. We demonstrate that MSA‐derived NPCs show changes of mitochondrial morphology and increased sensitivity to oxidative stress. MSA‐derived NPCs show no changes in *SNCA* expression and no spontaneous α‐synuclein inclusion formation but feature nuclear dislocation of the protein.

## Materials and methods

### iPSC generation and characterization

Fibroblasts were obtained from skin biopsies of two MSA patients and an age‐matched healthy control (Table S1) at the Department of Neurology, Medical University of Innsbruck with the appropriate consent and ethics approval. Cells were transduced with CytoTune™ 2.0 Sendai reprogramming vectors (A16517, Thermo Fisher Scientific) encoding for the classical Yamanaka’s factors (Klf4, Oct4, Sox2 and c‐Myc). The clearance of the Sendai virus (SeV) vector used for the somatic reprogramming was proven in the iPSCs cultures by quantitative RT‐PCR (qRT‐PCR) and immunocytochemistry and according to manufacturer’s instructions. Karyotyping was performed for each iPSC line by classical G‐banding at the Division of Human Genetics, Medical University of Innsbruck. Cells in metaphase arrest, induced by cocamide solution (0.1 µg/ml) were fixed, and Giemsa staining was performed. Chromosomes were analysed in at least 20 metaphases per cell line.

To confirm the pluripotency of the generated iPSCs, differentiation into the three germ layers (ectoderm, endoderm and mesoderm) was performed using StemMACS™ Trilineage Differentiation Kit (Miltenyi) according to the manufacturer’s protocols. Cells were analysed by immunocytochemistry at day 7 after initiation of the differentiation.

### NPCs generation and isolation

For neural induction, 24 h after seeding, iPSCs were cultured in neural induction medium ( 1x NEAA (Thermo Fisher Scientific), 1x Pen/Strep (Thermo Fisher Scientific), 1x B27 (‐Vit A) (Thermo Fisher Scientific), 10 µM SB431542 (StemGent), 250 nM LDN193189 (StemGent), 100 nM RA (Sigma), 25 µg/ml insulin (Sigma), and 1x β‐Mercaptoethanol (Thermo Fisher Scientific) in DMEM/F‐12 Glutamax (Thermo Fisher Scientific). Seven days after neural induction, PSA‐NCAM expressing NPCs were sorted by fluorescence‐activated cell sorting (FACS). Sorted NPCs were further cultured on Geltrex (Thermo Fisher Scientific) coated plates in DMEM/F‐12 Glutamax medium with 1x NEAA, 1x P/S, 1x N2 (Thermo Fisher Scientific), 10 ng/ml FGF (Sigma), 10 ng/ml EGF (Sigma) and 10 µg/ml Heparin (Sigma). The sorted and expanded NPCs were characterized by immunocytochemistry (ICC) for the expression of progenitor, neuronal, and glial markers.

### mRNA extraction and quantitative real‐time PCR (qRT‐PCR)

Messenger RNA (mRNA) was isolated from the cells using Dynabeads Oligo (dT)25 (Thermo Fisher Scientific) following the manufacturer’s protocol. Reverse transcription was performed using the High‐capacity cDNA Reverse Transcription Kit (Thermo Fisher Scientific). Control cDNA from hESC line 207 [34] was provided by Dr Nat.

The levels of gene expression were assessed by qRT‐PCR using the iTaq™ Universal Probes Supermix (BioRad) and Gene Expression Assays (Thermo Fisher Scientific, Table S2). RT‐PCR quantification was performed using Bio Rad CFX96 (BioRad*)*. Each experiment was run in two technical replicates. The results were normalized to the housekeeping gene glyceraldehyde‐3‐phosphate dehydrogenase (GAPDH) and expressed as ΔCt for each cell line (mean ± SD).

### Immunocytochemistry

Cells were washed with Dulbecco’s phosphate buffered saline (DPBS) and fixed with ice cold 4% paraformaldehyde (PFA) for 10 min. Cells were permeabilized with 0.2% Triton X‐100 in PBS (PBS‐T 0.2) at room temperature (RT). After blocking with the blocking solution (10% normal serum, 1% bovine serum albumin (BSA) in PBS‐T 0.1%), the cells were incubated overnight at 4°C with the primary antibody (Table S3) diluted in blocking solution. Next, cells were incubated with the secondary antibody (Table S3) diluted in 2% BSA in PBS‐T 0.1% for 90 min at room temperature. Counterstaining was done with 4',6‐Diamidino‐2‐Phenylindole (DAPI, Thermo Fisher Scientific) and final mounting with Fluoromount‐G® (Southern Biotech).

### Fluorescence Activated Cell Sorting (FACS)

Cells were harvested with accutase for 5 min at 37°C. After centrifugation, the pellet was re‐suspended in medium containing 10 µM of ROCK inhibitor (Stemcell Technologies). The cells were incubated with the antibody of interest (Table S4) at 4°C for 10 min, followed by one washing step with Buffer A (0.5% BSA, 2 mM ethylenediaminetetraacetic acid in DPBS) and centrifugation for 5 min at 300 g. Finally, the cells were resuspended in Buffer A and transferred to FACS tubes (Falcon). Settings for the appropriate sorting of PSA‐NCAM‐positive cells were established using the respective isotype controls. The cell sorting was performed at the FACS Facility of the Medical University of Innsbruck with the support of Dr Sieghart Sopper.

### Exposure to exogenous oxidative stress

NPCs at P3 were seeded in 24 well plates at a density of 10^5^ cells per well. After 24 h, to induce oxidative stress, the cells were exposed to different concentrations (0 mM, 0.5 mM, 1 mM, 5 mM) of Luperox® (Merk) for 2 h. Three wells per condition were analysed in at least four independent experiments.

### Detection of ROS

Measurement of the intracellular superoxide radical generation by the formation of a dark blue formazan deposit resulting from superoxide‐mediated reduction of NBT (nitroblue tetrazolium chloride, Roche Applied Sciences) was performed as previously shown [35, 36]. After exposure to Luperox, the cells were incubated for 30 min at 37°C with NBT (1:100) in DMEM‐F‐12/GLUTAMAX. The staining step was followed by two washes in DPBS and fixation for 10 min with cold 4% PFA at room temperature. After fixation of the cells, they were washed twice with PBS and then counterstained with DAPI (1 mg/ml) diluted in PBS (1:1000).

### Image analysis

Random optical fields in three independent replicates per treatment and cell line, and in at least three separate experiments were microphotographed with a 40x objective of a DMI 4000B Leica inverse microscope, provided with digital camera DFC300 FX and LAS V3.8 software (Leica). The images were blinded and further analysed by counting the total number of cells (DAPI staining) and the number of ROS‐positive and cleaved caspase‐3‐positive cells. The results for each cell line and treatment were presented as mean percentage of positive cells for every single experiment. The latter was calculated as the average of the three replicates in the experiment for cell line and treatment.

### High‐resolution respirometry

High resolution respirometry was performed at OROBOROS Instruments (Austria) according to a standard substrate‐uncoupler‐inhibitor titrations (SUIT)‐008 D025 protocol (https://bioblast.at/index.php/SUIT‐008_O2_ce‐pce_D025). Cell suspensions were transferred into calibrated Oxygraph‐2k 2 ml‐chambers. Oxygen polarography was performed at 37 ± 0.001 °C (electronic Peltier regulation) in O2k‐chambers. Oxygen concentration (μM), as well as oxygen flux per cell (pmol O_2_.s^−1^·cell^−1^) was recorded in real time using DatLab software.

Shortly, routine respiration was measured after the stabilization of the system. After permeabilization of the cells with digitonin (Dig), nonphosphorylating LEAK‐respiration (CI*_L_*) was induced by adding the CI‐linked substrates pyruvate (5 mM) and malate (0.5 mM). Subsequently, OXPHOS capacity of CI‐linked activity (CI*_P_*) was measured after addition of a saturating concentration of ADP (2.5 mM). Cytochrome C was added to assess the integrity of the mitochondrial outer membrane. To further evaluate the OXPHOS capacity, GDP and succinate were added (CI&II*_P_*). Stepwise titration of the protonophore carbonyl cyanide m‐chloro phenyl hydrazone (CCCP, 0.5 μM steps) led to proton leakage through the inner mitochondrial membrane, and was used for the measurement of the capacity of the electron transfer system (ETS, CI&II*_E_*), representing the noncoupled state at optimum uncoupler concentration for the maximum oxygen flux. Subsequent inhibition of CI by rotenone (0.5 μM) provided measurement of CII‐linked ETS capacity (CII*_E_*). To control for other oxygen‐consuming processes, CIII was inhibited by Antimycin A. The resulting residual oxygen consumption (ROX) reflected oxygen consumption from undefined sources and was subtracted from mitochondrial respiratory states [37]. ROX was very low, demonstrating the mitochondrial origin of oxygen consumption. Oxygen concentration in the chambers was kept high enough to avoid oxygen limitation of respiration (more than 150 μM O_2_ until the end of CI&II*_P_*). All reagents used for high‐resolution respirometry were purchased from Sigma‐Aldrich (St. Louis, Missouri, US). Cell‐specific oxygen fluxes were compared in different substrate and coupling states after correction for ROX. Flux control ratios (FCR) were calculated by dividing fluxes in all respiratory states of the SUIT protocol by CI&II‐linked ETS capacity taken as a common reference state [38]. Flux control factors (FCF) express the change of flux in a single step of the SUIT protocol normalized to a reference state with higher flux [37]. Stimulation of ADP‐saturated OXPHOS capacity, P, by optimum uncoupler concentration yields ETS capacity, E. The corresponding FCF is the apparent excess ETS capacity calculated as (E‐P)/E = 1‐P/E. The respiratory acceptor control ratio (RCR = P/L) was obtained in the CI‐linked substrate state. For statistical analysis RCR was transformed to its respective FCF, which is the OXPHOS coupling efficiency calculated as (P‐L)/P = 1‐L/P. The FCF for CI‐linked substrates stimulating CII‐linked respiration was measured as 1–CII/CI&II, in the ETS state in the SUIT protocol. The corresponding FCF for the CII‐linked substrate was calculated as 1–CI/CI&II, determined in the OXPHOS state in the SUIT protocol. Two replicates per cell line were analysed and the mean value per condition was used for the statistical analysis to compare the groups.

### Statistical analysis

For statistical analyses we used GraphPad Prism 8 Software. Comparisons between groups were done by t‐test, one– or two‐way ANOVA followed by post hoc analysis, depending on the dataset as indicated. A p‐value lower than 0.05 was considered significant.

## Results

### Cell model characterization

**Figure 1:**
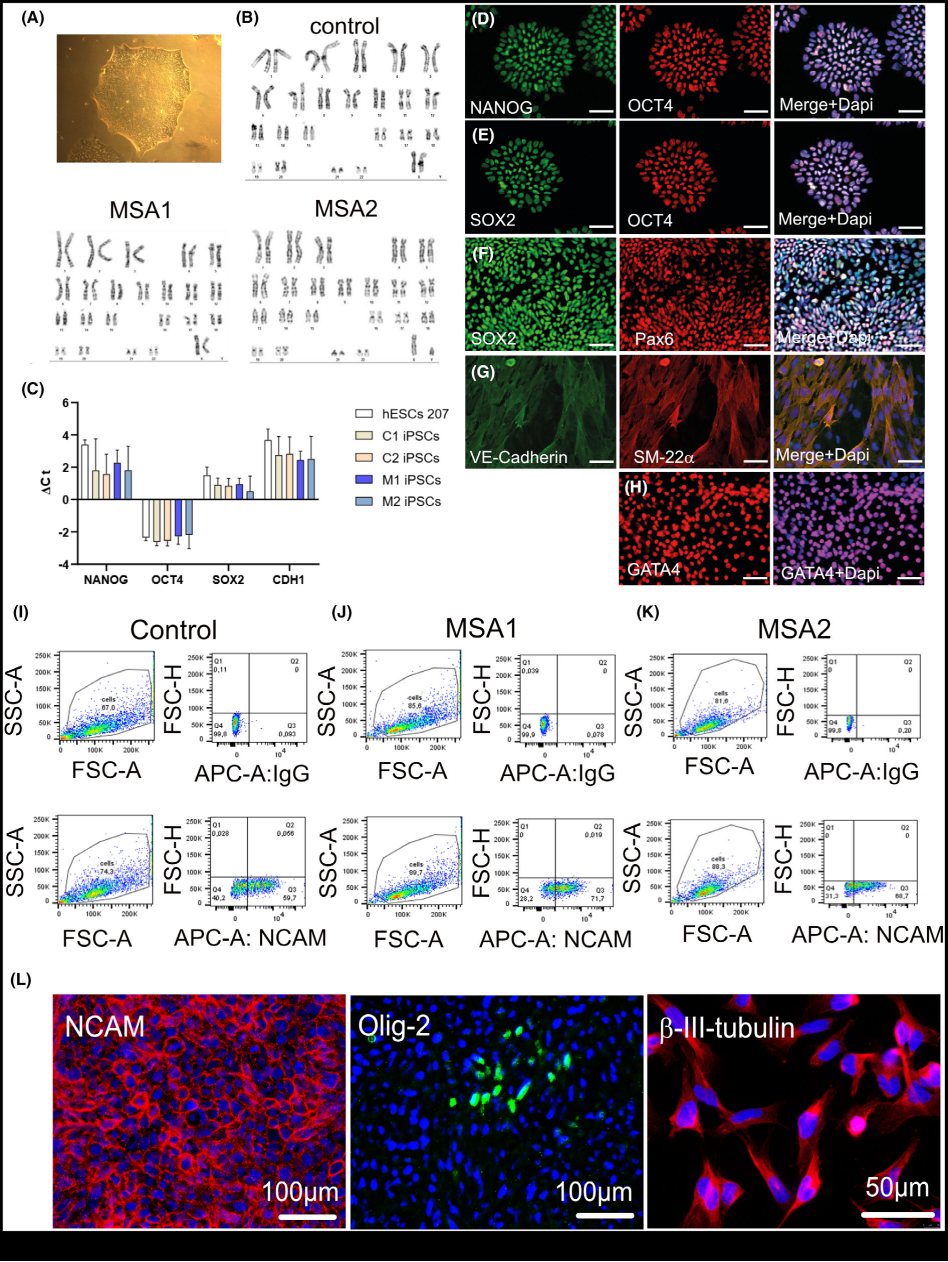
Generation and characterization of iPSCs and NPCs. **(A)** iPSCs colony of a validated MSA1 line **(B)** Representative karyotypes of iPSC lines. **(C)** Histogram depicting the ΔCt of pluripotent genes iPSC lines (C = control; M = MSA; hESC, human embryonic stem cells used as a reference)). Data are presented as Mean ± SD. **(D, E)** Expression of pluripotency markers NANOG, OCT4 and SOX2 in the generated iPSCs. **(F)** Ectoderm differentiation of iPSCs and PAX6 expression. **(G)** Mesoderm differentiation of iPSCs and VE‐Cadherin and SM22α expression. **(H)** Endoderm differentiation of iPSCs and GATA4 expression. Scale bars 100 µm. **(I‐K)** FACS plots demonstrating the sorted NCAM‐positive NPCs of control and MSA lines with the respective IgG control. These NPCs were used for the following experiments. **(L)** Expression of NCAM, Olig‐2, and β‐III‐tubulin in sorted NCAM‐positive NPCs. Counterstaining with DAPI (blue)

Two iPSC lines from two MSA‐P patients (MSA1, MSA2) and two iPSC lines from an age‐matched healthy control (C1, C2) were generated, characterized and used for the differentiation and further analysis of MSA and control NCAM‐positive NPCs (Figure 1). Skin fibroblasts were reprogrammed with nonintegrative SeV reprogramming vectors encoding *Klf4*, *Oct4*, *Sox2* and *c‐Myc*. The generated iPSCs showed normal karyotype (Figure 1B) and expressed genes of pluripotency at similar levels to hESCs (Figure 1C‐E). Furthermore, the pluripotency of the generated lines was demonstrated by differentiation towards ectodermal, mesodermal and endodermal fate (Figure 1F‐H). All cell lines were SeV free after 20 passages and used for the differentiation and sorting of NCAM‐positive NPCs ((Figure 1I‐K). No differences in the differentiation and yield of NCAM‐positive NPCs were identified between the control (40 ± 16%, n = 7 independent differentiation and sorting experiments) and MSA (51 ± 28%, n = 6 independent differentiation and sorting experiments) lines. The sorted NCAM‐positive NPCs were kept in culture and characterized by immunocytochemistry. In all cases, the NPCs expressed NCAM and β‐III‐tubulin (Figure 1L). A few cells were positive for Olig‐2 (Figure 1L), but no immunoreactivity for GFAP was detected (not shown).

### Mitochondrial elongation in MSA‐derived NPCs is not linked to changes in respiration

**Figure 2:**
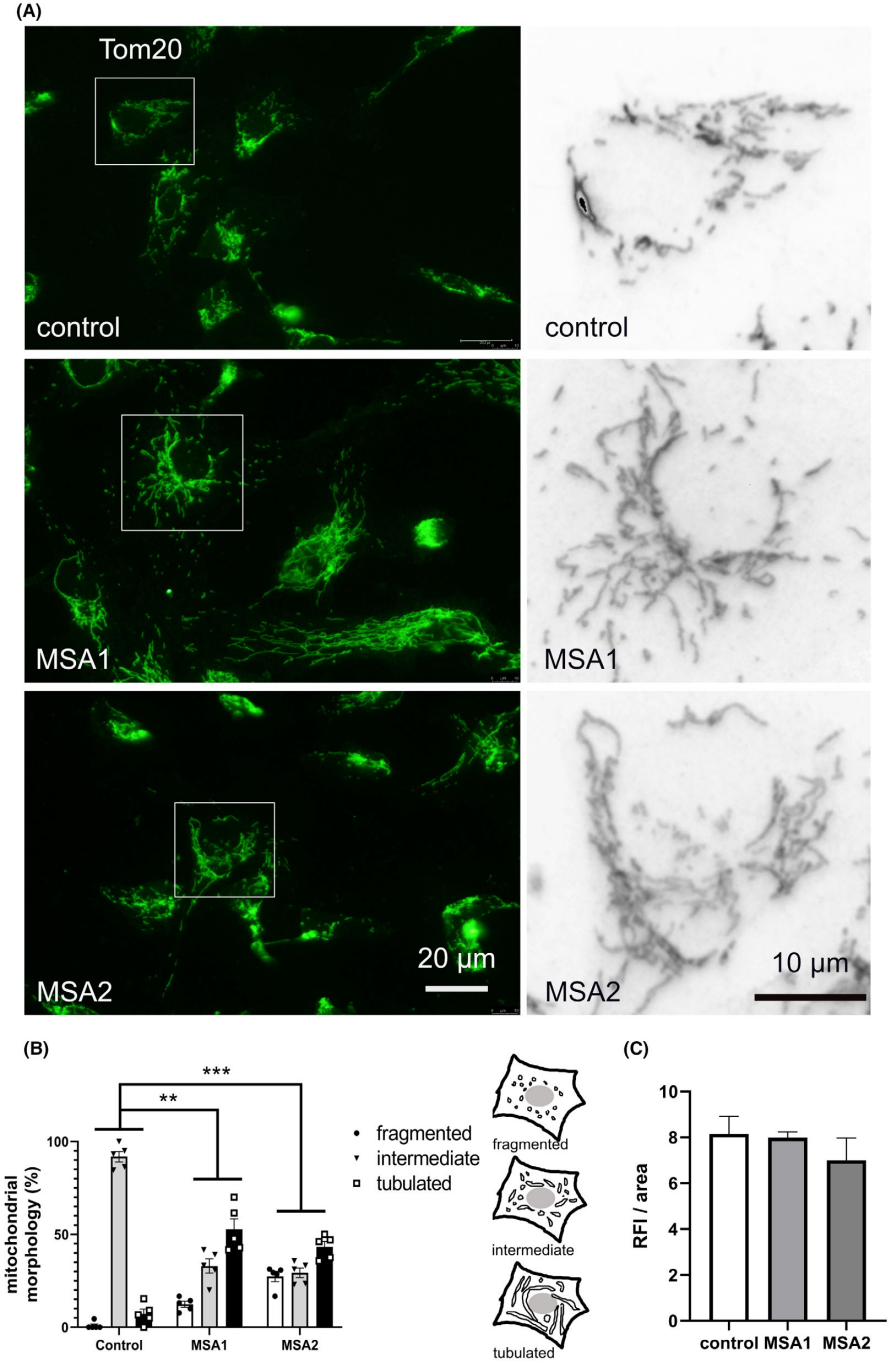
Shift of the mitochondrial morphology in MSA NPCs. (**A**) Mitochondria were visualized with Tom20 immunofluorescence in control and MSA‐derived NPCs. Insets are shown at higher magnification on the right. (**B**) The mitochondrial morphology was scored as previously described [42] in five samples per line: fragmented – mainly short and round; intermediate – round and short tubulated; tubular – long with higher connectivity. Data are presented as mean ± SEM. Two‐way ANOVA with Tukey’s posthoc correction for multiple comparison. **P < 0.01; ***P < 0.001. (**C**) The amount of mitochondria per cell was estimated by measuring the relative fluorescence intensity (RFI) per cell area using ImageJ. Data are presented as mean ± SEM. One‐way ANOVA comparison between the groups showed no significant differences (*P* = 0.578)

**Figure 3:**
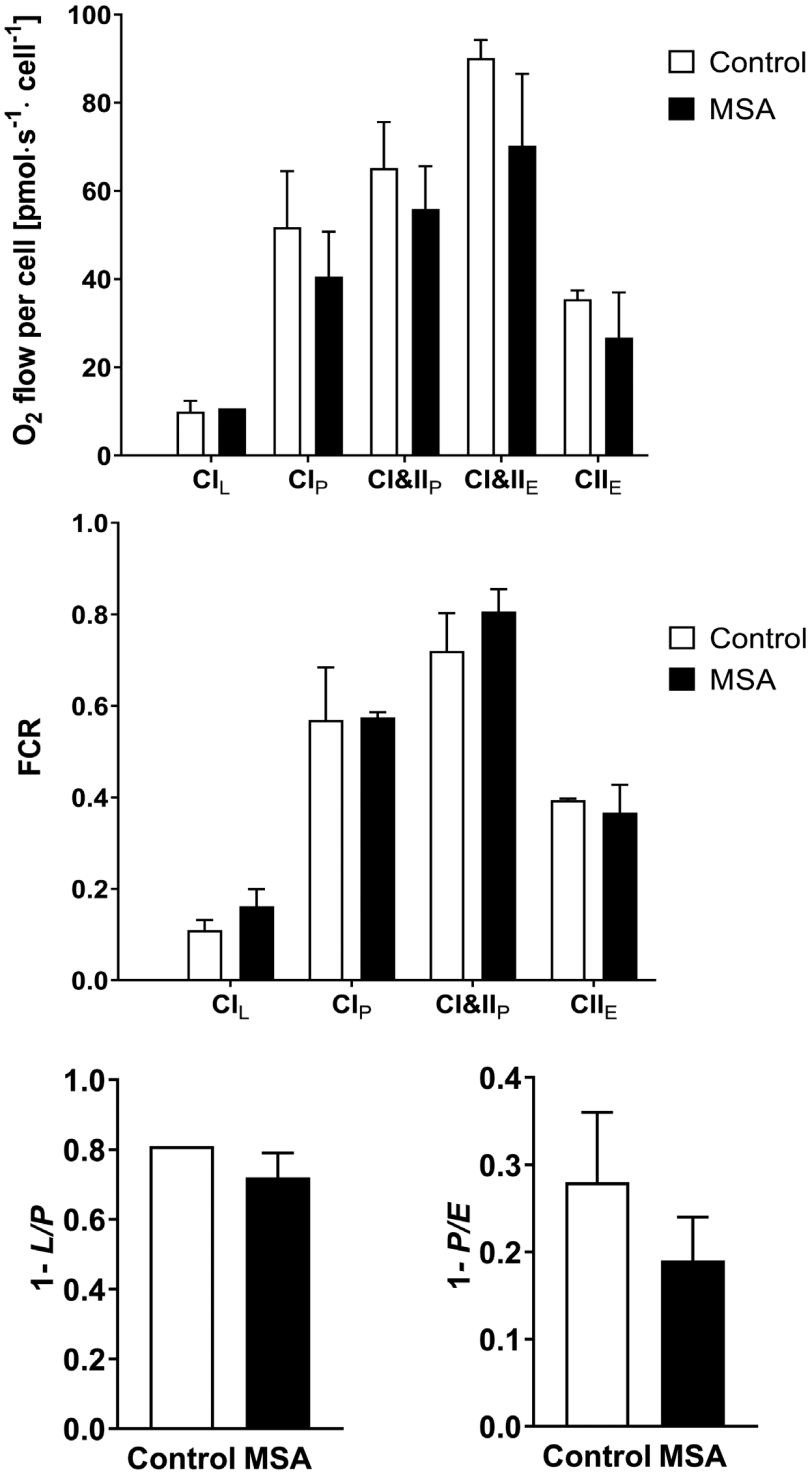
High resolution respirometry of MSA and control NPCs. (**A**) Absolute oxygen consumption per cell of control and MSA NPCs including CI‐linked (CI_L_) LEAK state after addition of CI substrate without ADP; CI‐linked (CI_P_) and CI&II‐linked (CI&II_P_) OXPHOS capacity as well as CI&II‐linked (CI&II_E_) electron transfer system (ETS) capacity and CII‐linked ETS capacity after addition of an uncoupler (CII_E_). (**B**) Flux control ratios (FCR) for all respiratory states referenced to CI&II_E_ ETS capacity. (**C**) Flux control factor (FCF) representing the OXPHOS coupling efficiency (1‐L/P); (**D**) FCF representing the excess of ETS capacity (1‐P/E). Data are presented as mean ± range. No group differences were observed between the single parameters by two‐way ANOVA or t‐test

Mitochondrial deficits have been suggested to play a role in MSA pathogenesis [10, 15, 39, 40, 41], therefore we first focussed on the mitochondrial morphology and function in NPCs. We imaged the general morphology and distribution of mitochondria by Tom20 immunocytochemistry (Figure 2A). Mitochondrial morphology was scored as previously suggested by Rambold and co‐workers [42]. We identified a redistribution from predominant intermediate phenotype in control cells towards more tubulated mitochondria in MSA cells (Figure 2B). We then estimated the relative area of mitochondria per cell in the different cell lines but detected no significant differences (Figure 2C). To explore further whether the observed changes in mitochondrial morphology are reflected by their respiratory function, we used high resolution respirometry. This method provides high sensitivity to measure oxygen flux even at 1pmol/s per cm^3^ in small samples and is offered for diagnostic tests of mitochondrial function in cell culture [43]. The extensive high resolution respirometry analysis identified no significant differences in the respiration of MSA vs control NPCs at any step of the coupling and substrate modulation to measure OXPHOS and ETS capacities (Figure 3).

### Oxidative stress conditions reveal higher susceptibility of MSA‐derived NPCs

**Figure 4:**
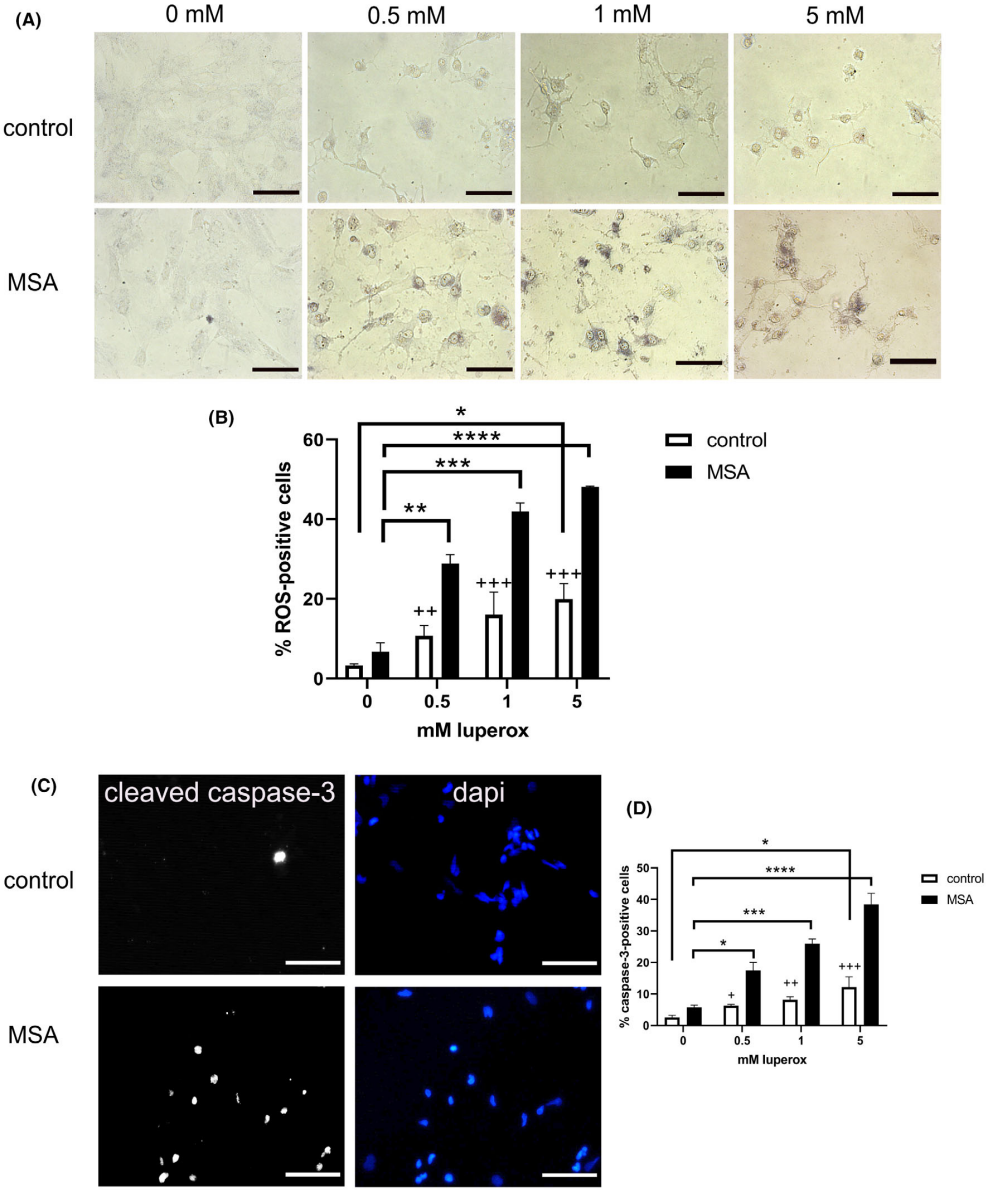
Exogenous oxidative stress in NPCs. (**A**) Intracellular ROS production (dark blue staining) after exposure to increasing concentrations (0‐5 mM) of luperox in control and MSA NPCs. Scale bars, 50 µm. (**B**) Percentage of cells with increased dark blue staining (ROS) after exposure to different luperox concentrations. Data are presented as mean ± range. Data were analysed with two‐way ANOVA followed by Sidak’s posthoc multiple comparison test. Crosses indicate the comparison of control and MSA NPCs exposed to the same concentration of luperox: ++*P* < 0.01; +++*P* < 0.001. *P < 0.05; **P < 0.01; ***P < 0.001; ****P < 0.0001. (**C**) Representative images of cleaved caspase‐3 activation in control and MSA NPCs exposed to 5 mM luperox. Scale bars, 100 µm. (**D**) Percentage of cells with positive cleaved caspase‐3 staining in the nucleus. Data are presented as mean ± range. Analysis was performed with two‐way ANOVA followed by Sidak’s posthoc multiple comparison test. Crosses indicate the comparison of control and MSA NPCs exposed to the same concentration of luperox: +*P* < 0.05; ++*P* < 0.01; +++*P* < 0.001. *P < 0.05; ***P < 0.001; ****P < 0.0001

Next, we sought to understand whether the morphological changes of mitochondria in MSA NPCs might indicate an adaptation mechanism of diseased cells to cope with energy stress and whether additional mild oxidative stress could be equally well compensated in MSA and healthy control cells. We used different doses of luperox, an organic peroxide, in order to set a threshold of oxidative stress in control and MSA NPCs. MSA NPCs showed an increased generation of ROS as compared to healthy control NPCs (Figure 4A). This increase was significant even after treatment with the lowest dose of luperox used here, while control cells showed significant levels of oxidative stress only after exposure to the highest dose (Figure 4B).

The increased production of ROS was paralleled by increased cleavage of caspase‐3, an effector caspase responsible for the last steps of programmed cell death. The percentage of cells positive for cleaved caspase‐3 was significantly increased in MSA NPCs after exposure to the lowest concentration of luperox, while control cells showed significant increase of caspase‐3 cleavage only after exposure to the highest used dose of luperox (Figure 4C,D).

### MSA‐derived NPCs show nuclear translocation of α‐synuclein without changes in the level of SNCA expression

**Figure 5:**
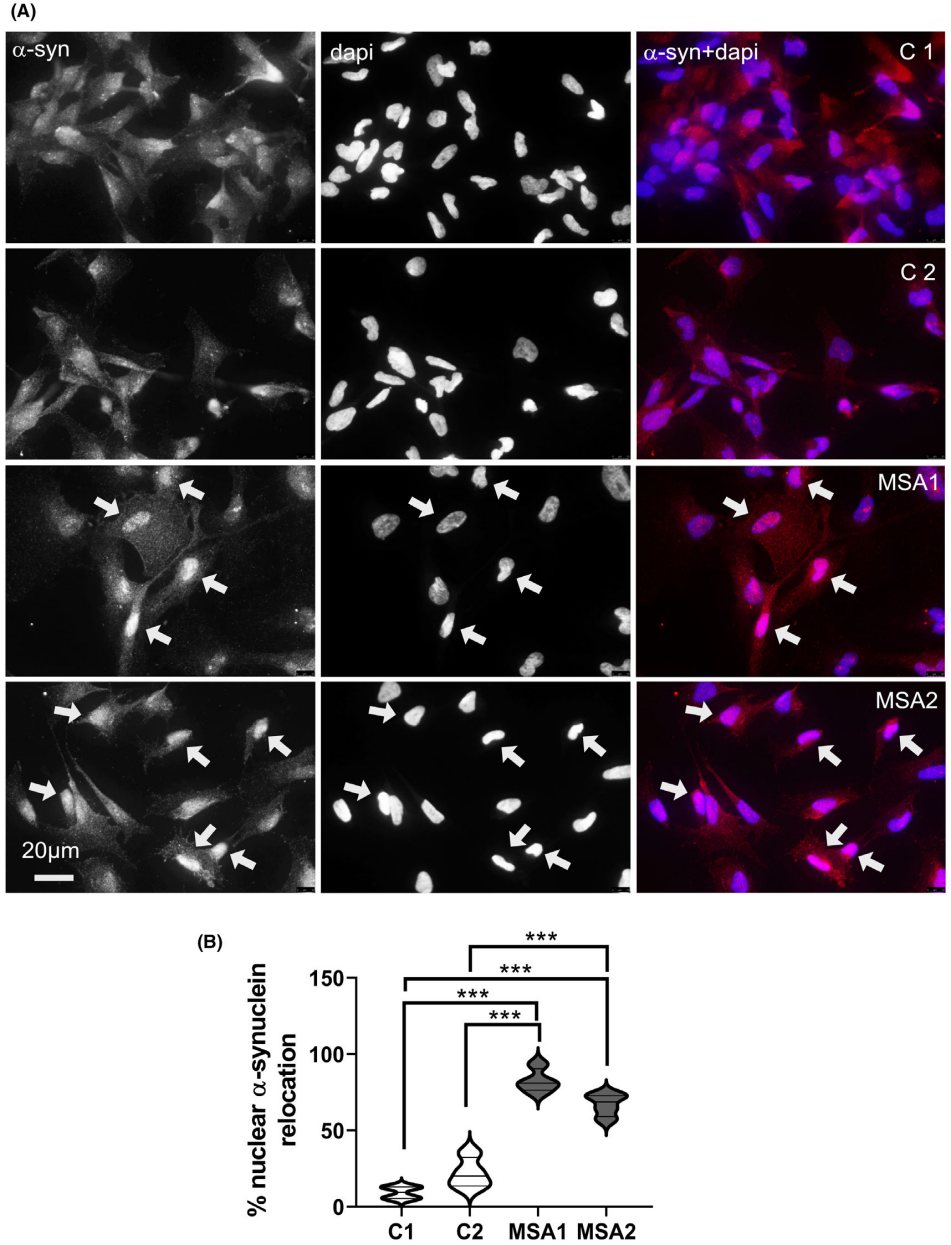
α‐synuclein expression in MSA and control NPCs. (**A**) Representative immunocytochemistry images show distribution of α‐synuclein in control NPCs C1 and C2 (cytoplasm and nucleus), and two MSA NPC lines (strong nuclear translocation (arrows)). (**B**) The percentage of NPCs with nuclear translocation of α‐synuclein in control and MSA lines in four samples per line. One‐way ANOVA with Tukey’s multiple comparison posthoc test. ***P < 0.001

Finally, we were interested to define, if α‐synuclein is expressed in NCAM‐positive NPCs and whether any disease‐related changes may be identified in this very early neural differentiation stage. qRT‐PCR analysis showed no difference in the level of SNCA expression in NCAM‐positive NPCs derived from MSA (ΔCt 7.6 ± 0.5, n = 3) and control (ΔCt 7.8 ± 0.5, n = 3). Interestingly, immunocytochemistry of control NPCs identified cells with a grainy pattern of positive staining throughout the cytoplasm and the nucleus (Figure 5A) as previously observed in mouse neuronal stem cells and different neural cell lines [44]. In the MSA NPCs, we observed translocation of α‐synuclein to the nucleus showing intense nuclear staining (Figure 5A). In both MSA cases, the α‐synuclein nuclear translocation at baseline was significantly higher as compared to control NPCs (Figure 5B). However, no α‐synuclein aggregates or signs of changed α‐synuclein phosphorylation were identified (not shown).

## Discussion

In this work, we generated NCAM‐positive NPCs from MSA patients and an age‐matched healthy control using the SeV reprogramming system for the generation of iPSCs and a dual SMAD inhibition protocol to generate NPCs. Due to its nonintegrating nature, the virus was diluted by passaging and did not interfere with our further experiments to assess disease‐related fingerprints in MSA‐specific iPSC‐derived NPCs. We show here morphological changes of the mitochondria and nuclear translocation of α‐synuclein in MSA‐derived NPCs without evident changes in respiration, cell viability or differentiation capacity at baseline. However, we identify for the first time a higher susceptibility of MSA‐specific NPCs to oxidative stress as compared to control NPCs, in a dose‐dependent manner.

As previously shown and corroborated in our experiments, PSA‐NCAM‐targeted sorting elicits highly homogeneous and expandable population of NPCs while retaining a typical morphology and molecular characteristics including expression of mostly neuronal (NCAM, β‐III‐tubulin) but also glial (Olig‐2) progenitor markers [45]. The *in vivo* behaviour of sorted human NCAM‐positive NPCs supports the broad differentiation potential of these cells toward various types of neurons, oligodendrocytes and astrocytes [45]. Therefore, this specific subpopulation of NPCs can inform about disease‐related deficits which may precede/trigger further dysfunction and pathology of neuronal and glial cells as is the case in MSA [21].

Our data indicate the existence of a pathological background in MSA‐derived NPCs, which is compensated functionally at baseline. Despite the changes in the mitochondrial phenotype in MSA‐derived NPCs there were no significant abnormalities in the respiration as measured by high resolution respirometry. Importantly, this method provides dynamic and highly sensitive measurement of metabolic flux in contrast to static determination of molecular components. Furthermore, it yields an integrative measure of the dynamics of complex coupled metabolic pathways and not simple monitoring of isolated enzyme activities [43]. This approach provides a more relevant estimate of the general functional state of the respiratory metabolic system, while large defects in single enzymes [40, 46] may be misleading and result in minor flux changes due to threshold effects. Mitochondrial elongation, which we describe here in MSA NPCs, has been previously reported to represent a putative protective adaptation mechanism against cellular damage [42, 47, 48]. Despite the changes in the morphology, we did not identify differences in the amount of mitochondria per cell, supporting the similar functional profile in high resolution respirometry of control and MSA lines. Interestingly, a previous study suggested that mitochondrial amount and respiratory capacity may be changed in MSA iPSC‐derived dopaminergic [40] suggesting a possible ‘sign of progression’ of the disease in a dish.

In line with this, we report here that the apparent balance of the redox homeostasis in MSA NPCs is easily disrupted by even low levels of exogenous oxidative stress. We selected oxidative stress for its relevance to the pathogenesis of MSA [49, 50, 51, 52] and for the possibility to precisely titrate the cellular exposure in vitro. Our data support the notion of higher susceptibility of MSA NPCs to exogenous stress. Despite the fact that the disease is mostly sporadic and no specific genetic aberrations are detected in GWAS [9], our data suggest that other stress response mechanisms may trigger cellular disbalance and the following pathology in MSA. We examined α‐synuclein expression, since this protein is considered a major player in the pathogenesis of MSA and other synucleinopathies. In these early developmental stages, we did not identify different levels of *SNCA* gene expression between control and MSA‐derived NPCs, supporting previous studies which concluded no causal relationship between the *SNCA* gene and MSA [27, 53]. However, we describe here a translocation of α‐synuclein to the nucleus of MSA NPCs. In control NPCs, we observed α‐synuclein expression through the soma and nucleus as described in earlier analyses [44, 54]. Intriguingly, we identified for the first time a nuclear translocation of α‐synuclein in MSA NPCs. Earlier studies by Outeiro and colleagues [44] showed that translocation of α‐synuclein to the nucleus may occur in cell lines under stress conditions, a finding which we confirmed in our control NPCs (Figure S1). For this reason, we believe that the nuclear translocation of α‐synuclein, like the mitochondrial morphological changes demonstrated in MSA NPCs, is yet another early sign of cellular stress. Previous experiments suggested that in cells overexpressing wild‐type α‐synuclein the nuclear location was associated with increased S129 phosphorylation and amyloid formation of α‐synuclein [44]. We were unable to identify similar changes in NPCs derived from MSA iPSCs, however the observed nuclear translocation in these cells may indicate an early event preceding α‐synuclein hyperphosphorylation and inclusion formation typical for MSA pathology [4]. The nuclear location of α‐synuclein has been associated with epigenetic modulation and DNA interaction resulting in transcriptional deregulation [44, 55, 56, 57], especially downregulation of cell cycle‐related genes, therefore supporting the notion that early changes in α‐synuclein cellular location in MSA NPCs may contribute to the observed increased susceptibility to exogenous stress.

All these facts let us hypothesize that in MSA, the initial α‐synuclein pathology is not related to misfolding, aggregate formation or prion properties of the protein, but rather associates with initial early relocation to the nucleus. Through the interactions of α‐synuclein with DNA and histones, it may trigger a preclinical condition which remains counterbalanced (no changes in respiration, no increased apoptotic cell death or ROS production at baseline). However, MSA NPCs are less prone to compensate exogenous stress which results in elicitation of the disease phenotype.

We need to acknowledge the limitations of this study related to the low number of lines used in our experiments linked to the restricted accessibility of cases suffering from the rare disease MSA and to its challenging diagnosis. At this stage, we are not in the position to increase the number of cases, however we believe that the reported data represent a very intriguing observation which deserves further extended evaluation in larger cohorts of patients (possibly in a multicentre study). A rare sporadic disease will necessitate a much larger effort to confirm or discard the observation made here, but we believe that through this report we can trigger the attention to events in MSA, that may precede α‐synuclein accumulation. Further studies in an extended cohort of MSA patients will be needed to expand the current observations and define the exact role of α‐synuclein nuclear translocation in MSA. However, based on the previous observations of Outeiro and colleagues [44] about the subcellular dynamics of α‐synuclein and associated transcriptional deregulation as well as supported by the presence of nuclear inclusions of α‐synuclein in MSA [4, 5, 58], we believe that our findings shed light on the early cellular dysfunction in MSA laying out a novel perspective of the approaches to study this devastating disease. In addition, further experimental data in differentiated neuronal and glial cultures derived from MSA iPSCs will further expand this line of research towards understanding the early stages of MSA pathogenesis.

## Ethical Approval

The research has been given ethics approval AN2016‐0105 362/4.16 by the local Ethics Committee.

## Author Contributions

MHV: acquisition and analysis of data; drafting and revising the manuscript. AHG: acquisition and analysis of data; revising the manuscript. FK: acquisition of clinical data and material; revising the manuscript. RD: acquisition and analysis of data; revising the manuscript. SB: conception; acquisition of clinical data and material, revising the manuscript. GKW: conception; acquisition of clinical data, interpretation of data; revising the manuscript. NS: conception and design; analysis, and interpretation of data; drafting and revising the manuscript. All authors read and approved the final manuscript.

### Peer Review

The peer review history for this article is available at https://publons.com/publon/10.1111/nan.12661.

## Supporting information


**Table S1.** Demographic data.
**Table S2.** TaqMan Gene Expression Assays used in the study.
**Table S3.** Antibodies used in ICC
**Table S4.** Antibodies used for FACS.
**Figure S1.** Nuclear translocation of α‐synuclein after exposure of NCAM‐positive NPCs derived from a healthy control to oxidative stress (luperox) supporting previous observations by Pinho et al 2019 [44].

## Data Availability

The data that support the findings of this study are available in the main body and the supplementary material of this article.
