# Supplementary material for: Signs of early cellular dysfunction in multiple system atrophy
Source: Neuropathol Appl Neurobiol. 2020 Sep 17;47(2):268–82. doi: 10.1111/nan.12661 (PMC7891639; doi:10.1111/nan.12661)
Supplement: Supplementary file 1 — Table S1. Demographic data. Table S2. TaqMan Gene Expression Assays used in the study. Table S3. Antibodies used in ICC Table S4. Antibodies used for FACS. Figure S1. Nuclear translocation of α‐synuclein after exposure of NCAM‐positive NPCs derived from a healthy control to oxidative stress (luperox) supporting previous observations by Pinho et al 2019 [44]. [file NAN-47-268-s001.pdf]

**Signs of early cellular dysfunction in multiple system atrophy**

Marcos Herrera-Vaquero[1], Antonio Heras-Garvin[1], Florian Krismer[1], Roxana Deleanu[2], Sylvia Boesch[1], Gregor K. Wenning[1], Nadia Stefanova[1*]

## SUPPLEMENTARY DATA

| Case | Diagnostic certainty | Age at skin biopsy | Sex | Age at symptom onset | UMSARS | Hoehn & Yahr Parkinson's Stage | Diagnosis |
|---|---|---|---|---|---|---|---|
| MSA1 | Probable MSA | 54 | F | 46 | 59 | 4 | MSA-P |
| MSA2 | Probable MSA | 54 | M | 52 | 31 | 3 | MSA-P |
| Control | - | 53 | F | - | - | - | - |

*Supplementary table 1. Demographic data*

| Human gene name | TaqMan gene expression assay |
|---|---|
| CDH1 | Hs01023895_m1 |
| GAPDH | Hs02786624_g1 |
| KLF4 | Hs00358836_m1 |
| LIN28A | Hs00702808_s1 |
| MYC | Hs00153408_m1 |
| NANOG | Hs02387400_g1 |
| POU5F1 (OCT4) | Hs04260367_gH |
| SEV | Mr04269880_mr |
| SEV-CMYC | Mr04269876_mr |
| SEV-KOS | Mr04421257_mr |
| SEV-KLF4 | Mr04421256_mr |
| SOX2 | Hs01053049_s1 |
| SNCA | Hs00240906_m1 |

*Supplementary table 2: TaqMan Gene Expression Assays used in the study.*

| Name | Produced in | Company | Cat No | Dilution |
|---|---|---|---|---|
| Oct3/4 | mouse | Santa Cruz | Sc-5279 | 1:100 |
| Nanog | rabbit | Abcam | Ab109250 | 1:200 |
| Sox 2 | rabbit | Abcam | Ab97959 | 1:200 |
| Pax6 | mouse | Santa Cruz | Sc-32766 | 1:100 |
| Gata4 | mouse | Santa Cruz | Sc-25310 | 1:100 |
| SM22α | rabbit | Abcam | Ab14106 | 1:200 |
| hVE Cadherin | goat | R&D | AF938 | 1:200 |
| Anti SeV | rabbit | MBL International Corporation | PD029 | 1:100 |
| CD56 (NCAM-1) | mouse | Invitrogen | MA106801 | 1:100 |
| Olig-2 | rabbit | Millipore | AB9610 | 1:500 |
| β-III-tubulin | rabbit | Abcam | Ab18207 | 1:1000 |
| GFAP | rat | Thermo Fisher | 13-0300 | 1:500 |
| Cleaved Caspase 3 | rabbit | Cell Signaling | 9661 | 1:100 |
| Tom20 | rabbit | Abcam | Ab186735 | 1:250 |
| Alpha-synuclein | mouse | Genetex | GTX21904 | 1:200 |
| Phospho S129 alpha-synuclein | rabbit | Abcam | Ab51253 | 1:1000 |
| Alexa 488 anti-mouse IgG | goat | Thermo Fisher Scientific | A11029 | 1:800 |
| Alexa 488 anti-goat IgG | donkey | Thermo Fisher Scientific | A11055 | 1:800 |
| Alexa 488 anti-rabbit IgG | goat | Thermo Fisher Scientific | A11034 | 1:800 |

| | | | | |
|---|---|---|---|---|
| Alexa 594 anti-mouse IgG | goat | Thermo Fisher Scientific | A11005 | 1:800 |
| Alexa 594 anti-rabbit IgG | goat | Thermo Fisher Scientific | A11037 | 1:800 |

*Supplementary table 3: Antibodies used in ICC*

| Name | Company | Cat No | Dilution |
|---|---|---|---|
| Anti-PSA NCAM-APC | Miltenyi | 130-093-273 | 1:11 |
| REA Control (S)-APC | Miltenyi | 130-113-434 | 1:11 |

*Supplementary table 4: Antibodies used for FACS.*

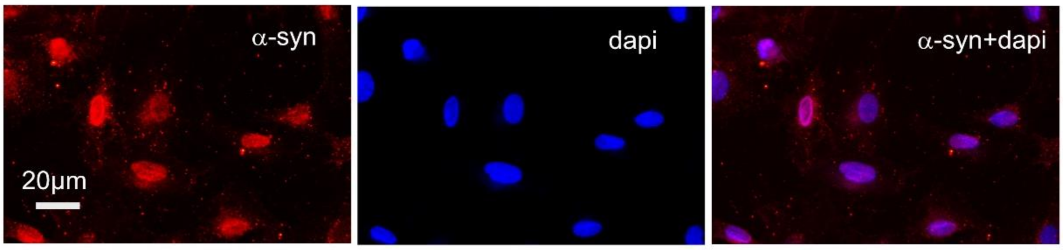


*Supplementary Figure 1. Nuclear translocation of α-synuclein after exposure of NCAM-positive NPCs derived from a healthy control to oxidative stress (luperox) supporting previous observations by Pinho et al 2019 [44].*
